# Effect of irradiation and bone marrow transplantation on angiotensin II-induced aortic inflammation in ApoE knockout mice

**DOI:** 10.1016/j.atherosclerosis.2018.07.019

**Published:** 2018-09

**Authors:** Jyoti Patel, Gillian Douglas, Alastair G. Kerr, Ashley B. Hale, Keith M. Channon

**Affiliations:** a Division of Cardiovascular Medicine, British Heart Foundation Centre of Research Excellence, Radcliffe Department of Medicine, John Radcliffe Hospital, University of Oxford, Oxford, OX3 9DU, UK; b Wellcome Centre for Human Genetics, University of Oxford, Oxford, OX3 7BN, UK; c Department of Physiology, Anatomy, and Genetics, University of Oxford, Oxford, OX1 3QX, UK

**Keywords:** Bone marrow transplantation, Angiotensin II, Aneurysm, Monocyte

## Abstract

**Background and aims:**

Angiotensin II (Ang II) infusion promotes the development of aortic aneurysms and accelerates atherosclerosis in *ApoE*^*−/−*^ mice. In order to elucidate the role of hematopoietic cells in these pathologies, irradiation and bone marrow transplantation (BMT) are commonly utilized. The aim of this study was to investigate the effects of irradiation and BMT on abdominal and thoracic aortic aneurysm formation and acute leukocyte recruitment in the aortic root and descending aorta, in an experimental mouse model of aortic aneurysm formation.

**Methods:**

*ApoE*^*−/−*^ mice were either lethally irradiated and reconstituted with *ApoE*^*−/−*^ bone marrow or non-irradiated. Following engraftment, mice were treated with Ang II to induce aortic inflammation and accelerate atherosclerosis.

**Results:**

Ang II infusion (0.8 mg/kg/day) in BMT mice resulted in reduced aortic aneurysms and atherosclerosis with decreased leukocyte infiltration in the aorta compared to non-BMT mice, when receiving the same dose of Ang II. Furthermore, the reduced aortic infiltration in BMT mice was accompanied by increased levels of monocytes in the spleen and bone marrow. A dose of 3 mg/kg/day Ang II was required to achieve a similar incidence of aneurysm formation as achieved with 0.8 mg/kg/day in non-BMT mice.

**Conclusions:**

This study provides evidence that BMT can alter inflammatory cell recruitment in experimental mouse models of aortic aneurysm formation and atherosclerosis and suggests that irradiation and BMT have a considerably more complex effect on vascular inflammation, which should be evaluated.

## Highlights

• Ang II infusion in bone marrow transplanted mice results in fewer aneurysms with decreased aortic leukocyte infiltration.
• Reduced aortic infiltration in bone marrow transplanted mice is accompanied by increased levels of monocytes in the spleen.
• Irradiation and bone marrow transplantation has complex effects on vascular inflammation which should be evaluated.

## 1 Introduction

Angiotensin II (Ang II) infusion into *ApoE*^*−/−*^ mice augments atherosclerotic plaque and abdominal aortic aneurysm (AAA) formation [1], via mechanisms causing increased monocyte-macrophage recruitment and vascular wall remodeling. Accordingly, Ang II-induced vascular inflammation can be studied by treating hyperlipidaemic mice with Ang II to investigate long-term, chronic inflammatory responses such as plaque formation, or short-term acute inflammatory processes such as cellular infiltration. To explore the role of hematopoietic cells in disease models, irradiation and bone marrow transplantation (BMT) have been used as a tool to generate chimeric mice, receiving donor bone marrow cells from transgenic mice harboring a genetic alteration in a functional pathway [2].

For BMT experiments, bone marrow cells are isolated from the donor mouse and injected (usually intravenously) into the recipient mouse, which has been irradiated to ablate its bone marrow cells.

Following successful BMT, the hematopoietic system is generally repopulated within 4 weeks [2,3]. The effect of BMT on the susceptibility to atherosclerosis has indicated that irradiation has more complex outcomes on plaque formation. In BMT studies in LDL-receptor deficient mice fed a high fat diet, aortic root lesions were greater in the BMT mice, while the lesions in the descending aorta were greater in the non-BMT mice [4]. In line with the a former observation, other BMT studies in *ApoE*^*−/−*^ mice have suggested that high levels of irradiation or fractionated irradiation accelerates the development of macrophage-rich, inflammatory atherosclerotic lesions in the carotid arteries [5,6]. However, the direct effects of irradiation and BMT on Ang II-induced aortic aneurysm formation and Ang II-induced atherosclerosis has not be examined.

Bone marrow transplantation (BMT) following irradiation is commonly used in experimental studies designed to investigate the specific contribution of BM-derived circulating cells to inflammatory disease processes. During previous studies investigating the formation of abdominal aortic aneurysms (AAA) induced by Ang II in *ApoE*^*−/−*^ mice that had undergone irradiation and BMT to generate chimeric mice with different gene expression in BM-derived cells *versus* host cells, we observed that irradiation and BMT itself affects the degree of AAA development and rupture in *ApoE*^*−/−*^ mice receiving *ApoE*^*−/−*^ bone marrow. In this current study, we further investigated the effects of BMT on aortic inflammatory disease and accelerated atherosclerosis in male *ApoE*^*−/−*^ mice treated with Ang II. Here, we report that aneurysm formation in BMT *ApoE*^*−/−*^ mice treated with Ang II was absent and required an increase in the dose of Ang II to achieve the same degree of aneurysm formation and rupture to that of non-BMT *ApoE*^*−/−*^ mice treated with Ang II, highlighting the need to interpret BMT and Ang II-induced AAA studies more critically.

## 2 Materials and methods

### 2.1 Mice

*ApoE*^*−/−*^ mice were housed in individually ventilated cages with 12 h light/dark cycle and controlled temperature (20 °C - 22 °C). Standard chow (B & K Universal Ltd, UK) and water were available *ad libitum*. All animal studies were conducted with ethical approval from the Local Ethical Review Committee and in accordance with the UK Home Office Animals (Scientific Procedures) Act 1986.

### 2.2 Bone marrow transplantation (BMT)

Mice were irradiated (2 × 5 Gy) and transplanted with bone marrow cells (5 × 10^6^) at the age of 10 weeks. As a control for the efficiency of the irradiation procedure and bone marrow transfer we performed rigorous testing to characterise and confirm chimerism. We used mouse strains with alternate CD45.1 or CD45.2 leukocyte antigens to allow ‘tracking’ of engrafted cells from the donor in the chimeric recipient after marrow ablation by irradiation. We confirmed that more than 95% blood cells in recipient mice are donor-derived 6 weeks after transplantation, with reconstitution of normal peripheral leukocyte counts and subset profiles confirming successful reconstitution (Supplementary Figure 1). Hereafter, BMT will indicate bone marrow transplantation, which is an all-inclusive term for the accompanying irradiation as well as the bone marrow reconstitution.

### 2.3 Ang II infusion, blood pressure recordings and aneurysm characterisation

For these studies, we used the Ang II infused *ApoE*^*−/−*^ C57/Bl6 mouse model developed in the laboratory of Daugherty et al. [1]. In this mouse model, suprarenal AAAs form in up to 80–85% of the cases. As indicated in Robinet et al., there is a low AAA incidence in Ang II infused female *ApoE*^*−/−*^ mice (∼20%), therefore we chose to study male *ApoE*^*−/−*^ mice that have between 80 and 100% AAA incidence [7]. Both sex hormones [8] and sex chromosomes [9] have been shown to effect AAA development in experimental AAA. Once, successful reconstitution was confirmed after 6 weeks of recovery, mice were infused with Ang II for 5 or 14 days. Systolic blood pressure was measured on day 2 – day 14 of Ang II or saline infusion. Briefly, BMT or non BMT male mice were anaesthetized with isoflurane by inhalation and osmotic mini-pumps (Alza Corp, USA) delivering Ang II (0.8, 1.5, or 3 mg/kg/day; Sigma-Aldrich, UK) were implanted subcutaneously. Systolic blood pressure was measured using an indirect method of blood pressure measurement in animals, using a non-invasive computerized tail-cuff system in conscious mice following a 1 week training period (Visitech BP2000, Visitech Systems, Inc., USA) following guidelines in Kurtz et al. [10]. We categorised aortic aneurysms as remodelled tissue in the supra-renal and/or thoracic regions of the aorta that contained thrombus of surviving mice at harvest (Type II-Type IV). Aortic aneurysm rupture was characterised by blood in the cavity and large thrombi in the aorta of mice that were found dead. ‘No aneurysm’ was characterised by the absence of any thrombi and the absence of large dilatation in the supra-renal and/or thoracic regions of the aorta of surviving mice at harvest. Maximal outer widths of suprarenal abdominal aortas were measured using Image Pro Plus software (Media Cybernetics, USA) by a researcher blind to the group assignment.

### 2.4 Atherosclerosis analysis

All animal atherosclerosis measurements were based on the recommendations in Daugherty et al. [11]. *ApoE*^*−/−*^ mice were fed a normal mouse chow diet (19.67% crude protein, 4.13% crude oil and 3.22% crude fibre; B & K Universal Ltd, UK) and atherosclerotic lesion size was assessed in paraffin-embedded aortic root sections stained with Masson-Goldner trichrome (Merck, Germany). Serial sections of 7 μm thickness were cut with a microtome (Leica RM2155; Leica Microsystems) from the first appearance of the tricuspid valves until valves were no longer visible and mounted onto adhesive poly-l-lysine coated slides (VWR, Leicestershire, UK). Approximately 60–80 sections were cut for each animal and anatomical markers were used to enable sections from 3 depths within the cusp region to be taken at the same depth in each animal for staining. The average lesion size was calculated from three sections taken at 100 μm intervals starting from the section showing all three aortic cusps. For immunohistochemical staining, serial sections of aortic roots were assessed for macrophage content using an anti-Galectin-3 antibody (BD Pharmingen, UK). As controls for positive staining, paraffin embedded liver sections were stained in parallel experiments for Galectin-3. For negative controls, one section on each slide for each animal was incubated with the respective isotype antibody to determine staining specificity. Aortic roots were visualised and imaged (coolSNAP-pro camera, Roper Scientific, Leica DMRBE microscope and the lesion area and Galectin-3 positive areas quantified from digitized microscopic images using Image-Pro Plus (Media Cybernetics, USA).

### 2.5 Flow cytometry

Descending aortas from the aortic arch to femoral bifurcations that did not contain a visible aneurysm were micro-dissected and digested in an enzyme solution containing 60 U/ml DNase I, 60 U/ml Hyalronidase, 450 U/ml Collagenase I and 125 U/ml Collagenase XI (all enzymes from Sigma-Aldrich, UK) at 37 °C as described in Galkina et al. [12]. A single cell suspension was prepared by passing aortic pieces through a strainer for subsequent flow cytometry staining. Isolated aortic cells were stained with monoclonal antibodies directed against CD45+, CD11b+, Ly6C+, Ly6G–(monocytes) and CD45+, CD11b+, Ly6C+, Ly6G+ (neutrophils) (BD Pharmingen) with appropriate isotype controls as described in Tieu et al. [13]. Absolute cell counts were performed by ratio to a known quantity of calibration beads added to each sample (CaliBrite, BD Pharmingen, UK). Data was acquired using a CyAn Analyser flow cytometer (Beckman Coulter Ltd, UK) and then analysed using Summit (Dako, UK) and FlowJo (Tree Star Inc, USA) software.

### 2.6 Statistical analysis

Briefly, animal sample size for each experiment was chosen based on literature documentation and previous well characterised experiments. Data are expressed as mean ± SEM as indicated. Statistical differences were measured using an unpaired Student's t-test, or one-way ANOVA with Dunnett's or Newman-Keuls multiple comparisons test. Normality was checked using the Kolmogorov-Smirnov test. A nonparametric test (Mann-Whitney) was used when data did not pass the normality test. % survival was calculated using the χ2-test and % incidence was calculated using a χ2-test and Fisher's exact test to compare aneurysm (including rupture) versus no aneurysm. A value of *p* ≤ 0.05 was considered statistically significant. Data analysis was performed using GraphPad Prism Software Version 7 (GraphPad, San Diego, CA).

## 3 Results

### 3.1 BMT results in reduced Ang II induced aortic aneurysms and an increasing dose of Ang II is required to achieve a similar incidence of aortic aneurysms to that of non-BMT mice

**Fig. 1:**
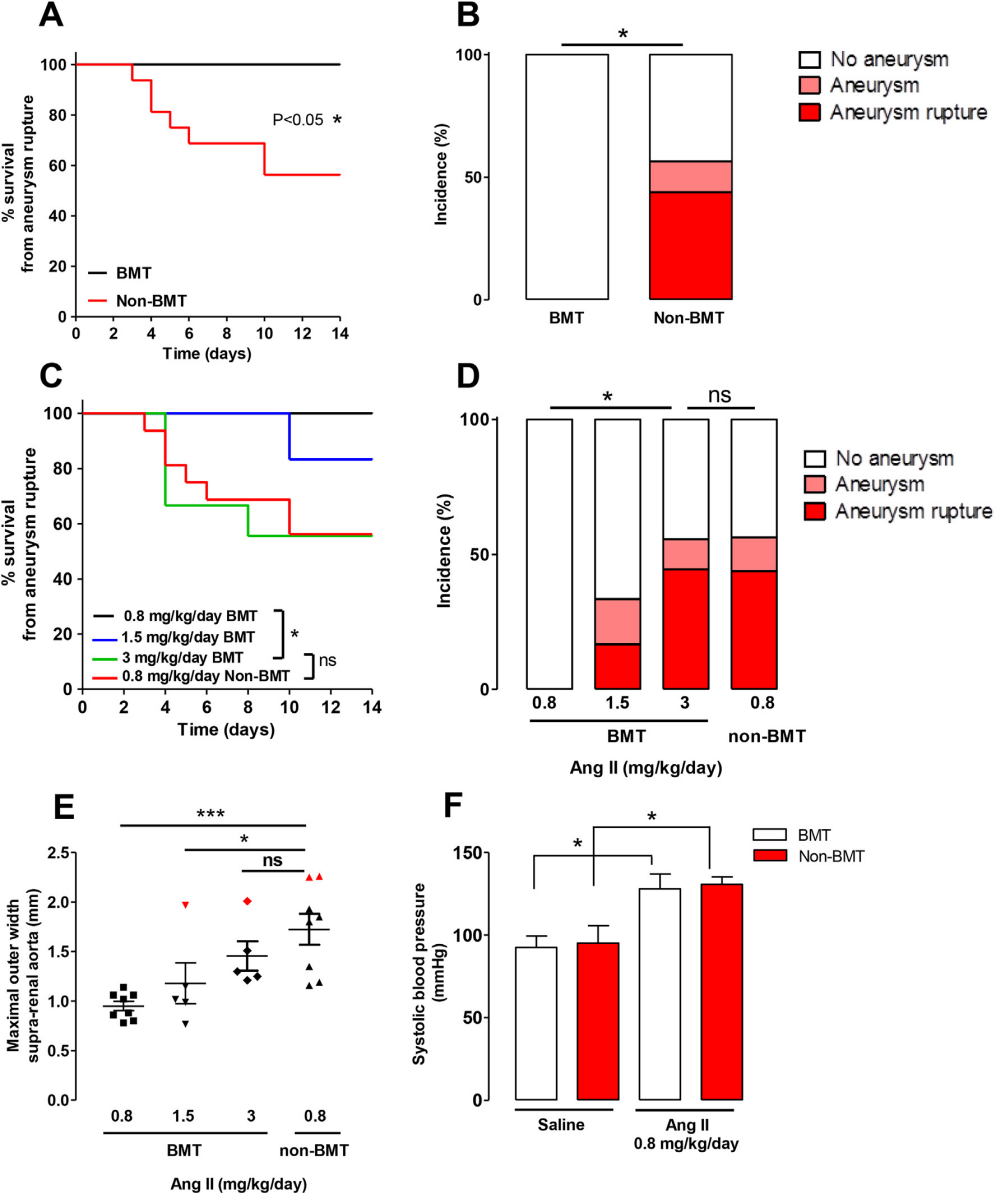
BMT reduces the incidence of Ang II-induced aortic aneurysm rupture in 0.8 mg/kg/day Ang II-treated *ApoE^−^^/^^−^* mice. (A) Survival curve of BMT and non-BMT mice during Ang II (0.8 mg/kg/day) infusion. All deaths were due to aortic rupture. (B) The incidence of Ang II-induced aortic aneurysms in BMT mice compared with non-BMT mice during Ang II (0.8 mg/kg/day) infusion. (C) Survival curve of BMT and non-BMT mice during Ang II infusion (0.8, 1.5 or 3 mg/kg/day). All deaths were due to aortic rupture. (D) The incidence of Ang II-induced aortic aneurysms in BMT mice compared with non-BMT mice during Ang II infusion (0.8, 1.5 or 3 mg/kg/day). (E) Maximal outer width of the suprarenal aorta of BMT mice compared with non-BMT mice during Ang II infusion (0.8, 1.5 or 3 mg/kg/day). Mice, which were classified as having thrombi containing aneurysms in the suprarenal region, are highlighted in red. (F) The systolic blood pressure of BMT and nonBMT mice infused with Ang II (0.8 mg/kg/day) over 14 days. *p < 0.05 in (A, C) calculated using the χ2-test (n = 8–16). *p < 0.05 in (B, D) calculated using the χ2-test and Fisher's exact test for aneurysm (including rupture) *vs.* no aneurysm. *p < 0.05 in (E) calculated by One-way ANOVA followed by the post Dunnetts multiple comparisons test (n = 5–8). *p < 0.05 in (F) calculated by One-way ANOVA followed by the Newman-Keuls Multiple Comparison Test (n = 3 for saline groups, n = 6 for Ang II groups). (For interpretation of the references to colour in this figure legend, the reader is referred to the Web version of this article.)

To determine the effect of BMT on the development of AAA, we infused Angiotensin II (Ang II) at 0.8 mg/kg/day using subcutaneous mini pumps in male *ApoE*^*−/−*^ mice that had undergone BMT and matched control non-BMT animals over 14 days. Between days 3 and 10 of Ang II infusion, 7 out of 16 non-BMT *ApoE*^*−/−*^ mice died from aneurysm rupture, but no deaths occurred in BMT mice, indicating that BMT may confer protection from Ang II-induced aortic aneurysm rupture (Fig. 1A). We also noted a 13% incidence of aneurysms at the study end point in surviving non-BMT *ApoE*^*−/−*^ mice, which were absent in BMT *ApoE*^*−/−*^ mice (Fig. 1B). In order to achieve a similar degree of aortic aneurysms in BMT mice, as observed in non-BMT mice treated with 0.8 mg/kg/day Ang II, we performed an Ang II dosing experiment in BMT mice for 14 days. We found that 3 mg/kg/day of Ang II in BMT mice were sufficient to induce aortic aneurysm rupture at a level with non-BMT mice receiving 0.8 mg/kg/day Ang II, in comparison to 0.8, or 1.5 mg/kg/day of Ang II in BMT mice (Fig. 1C). Additionally, the incidence of aneurysms in surviving mice at the end of the study was similar between 0.8 mg/kg/day Ang II in non-BMT mice and 3 mg/kg/day Ang II in BMT mice (Fig. 1D; 13% *versus* 11%). In contrast, treatment with 1.5 mg/kg/day resulted in a 16% incidence of aneurysm formation and 16% incidence of aneurysm rupture. In line with these findings, the maximal outer width of the suprarenal aorta was markedly increased in the non-BMT group receiving 0.8 mg/kg/day Ang II compared to the BMT group receiving 0.8 mg/kg/day Ang II, but no differences in width were observed between the BMT 3 mg/kg/day Ang II group and non-BMT group receiving 0.8 mg/kg/day Ang II (Fig. 1E).

Since Ang II infusion increases systolic blood pressure in mice and to determine whether BMT affects Ang II-mediated increases in blood pressure, we measured the systolic blood pressure in both groups via tail cuff. Ang II treatment increased systolic blood pressure in both BMT and non-BMT mice to the same extent in comparison to the saline control groups (Fig. 1F), demonstrating that protection from aneurysm formation in BMT mice occurs despite an increase in blood pressure and through mechanisms that are independent of Ang II-induced hypertension.

### 3.2 BMT results in reduced Ang II –mediated atherosclerosis in *ApoE*^*−/−*^ mice

**Fig. 2:**
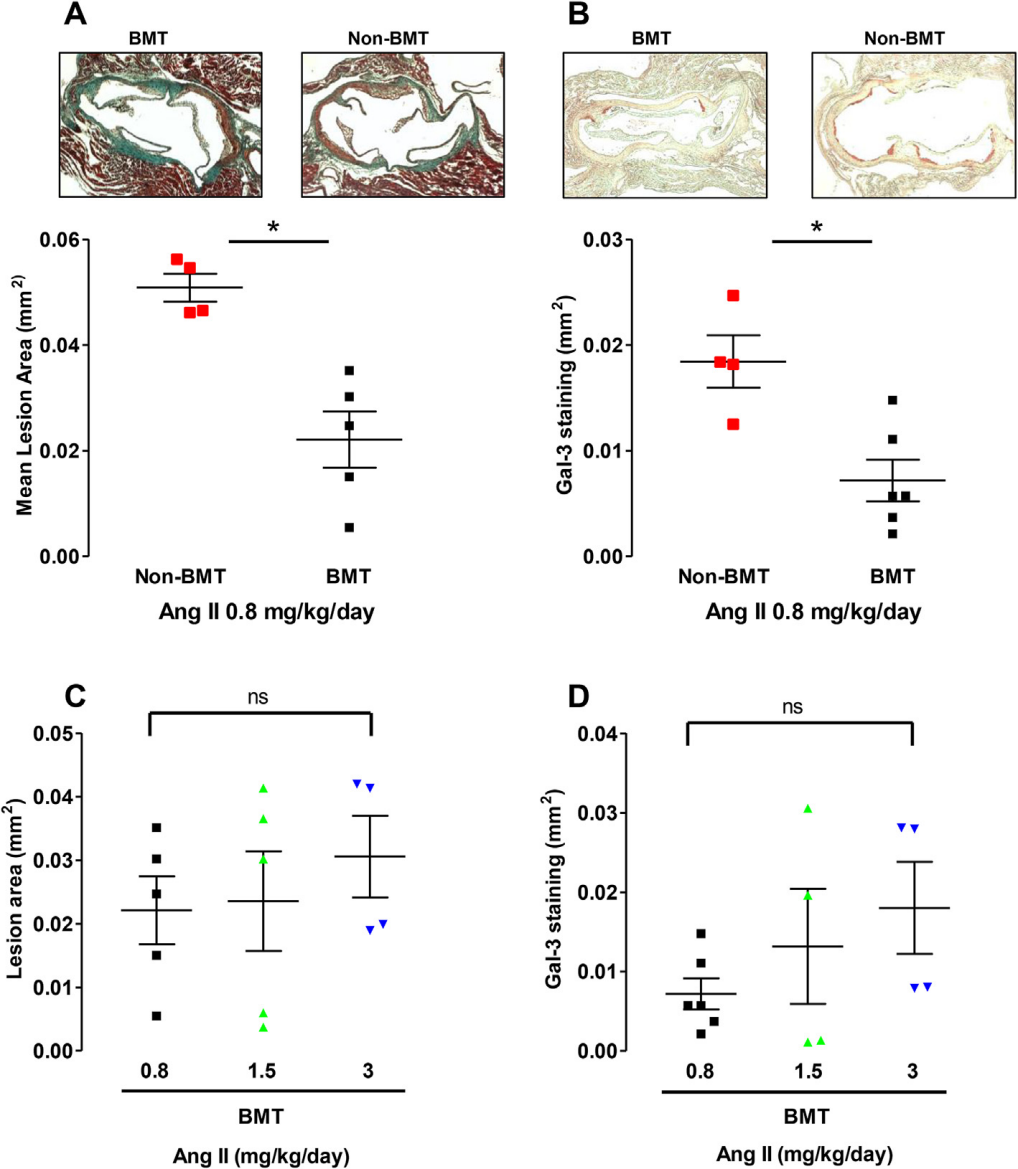
BMT reduces Ang II-mediated accelerated atherosclerosis and macrophage content in 0.8 mg/kg/day Ang II-treated *ApoE^−^^/^^−^* mice. (A) Atherosclerotic plaque in the aortic roots of 18-week old BMT and non-BMT mice following Ang II (0.8 mg/kg/day) infusion for 14 days. Mice were kept on a chow diet. Microscopy of Massons trichrome stained aortic root lesions. (B) Galectin-3 positive macrophage content in the aortic roots of 18-week old BMT and non-BMT mice following Ang II (0.8 mg kg−1 per day) infusion for 14 days. Microscopy of Galectin3 (Gal-3) stained aortic root lesions. (n = 4–6 per group) *p < 0.05. (C) Atherosclerotic plaque in the aortic roots of 18-week old BMT mice following Ang II infusion II (0.8, 1.5, or 3 mg/kg/day) for 14. days. (D) Galectin-3 positive macrophage content in the aortic roots of 18-week old BMT mice following Ang II infusion (0.8, 1.5, or 3 mg/kg/day) for 14 days.

Ang II treatment also accelerates plaque progression and leukocyte infiltration in lesions in *ApoE*^*−/−*^ mice [1,14,15]. Hence we sought to determine whether BMT and Ang II infusion would alter atherosclerosis in BMT and non-BMT *ApoE*^*−/−*^ mice following Ang II infusion at 0.8 mg/kg/day for 14 days. *ApoE*^*−/−*^ mice were kept on a chow diet for the entire period of the study. BMT in *ApoE*^*−/−*^ mice treated with Ang II (0.8 mg/kg/day) significantly reduced both atherosclerotic plaque formation in the aortic root (Fig. 2A) and reduced plaque macrophage content, quantified by Galectin-3-positive macrophage immunostaining (Fig. 2B) compared with non-BMT *ApoE*^*−/−*^ mice treated with the same dose of Ang II. Increasing the dose of Ang II induced comparable levels of plaque size (Fig. 2C) which was largely driven by macrophage infiltration (Fig. 2D) between non-BMT mice receiving 0.8 mg/kg/day Ang II and BMT mice receiving 3 mg/kg/day of Ang II.

### 3.3 Altered monocyte infiltration after BMT and 0.8 mg/kg/day Ang II infusion

**Fig. 3:**
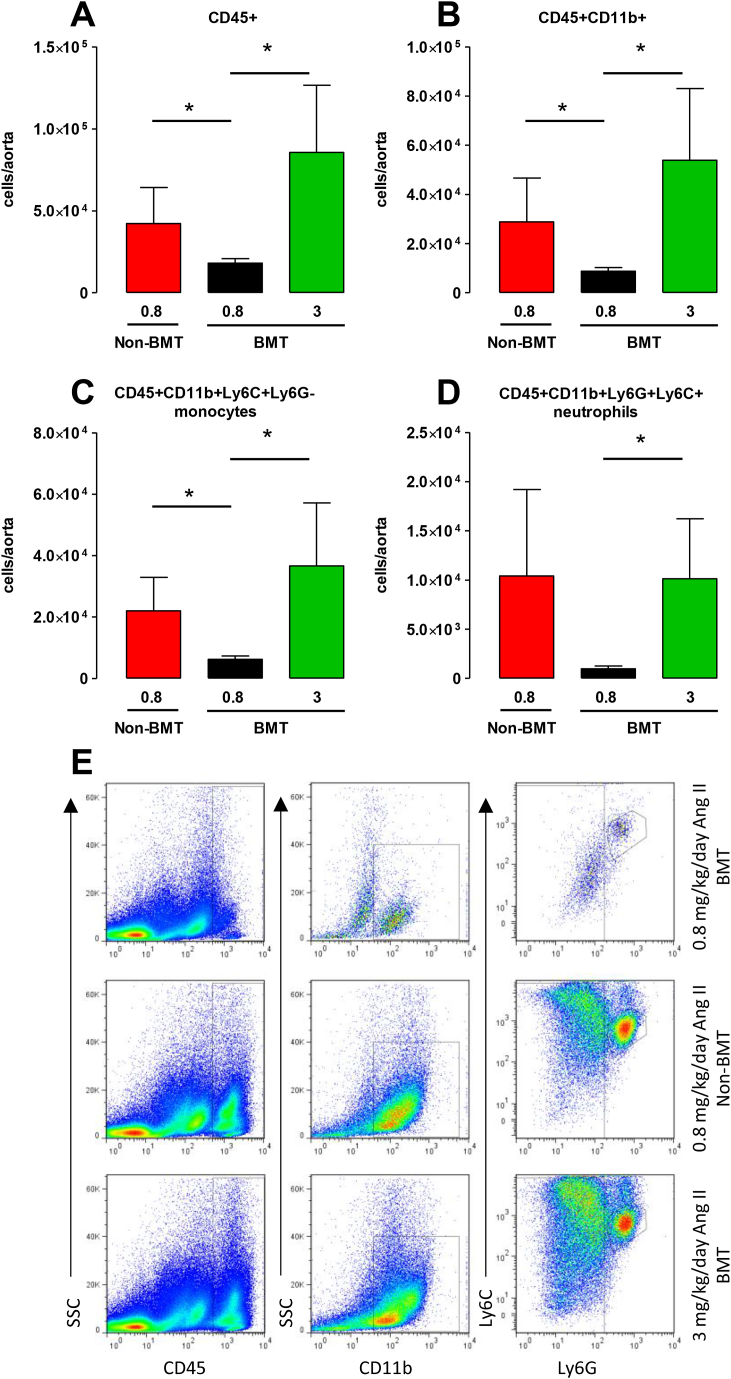
BMT decreases aortic leukocyte trafficking in 0.8 mg/kg/day Ang II-treated *ApoE^−^^/^^−^* mice. Flow cytometric analysis of aortic leukocytes in BMT and non-BMT mice that received Ang II infusion at 0.8 or 3 mg/kg/day for 5 days. Quantification of the numbers of (A) CD45 ^+^ cells, (B) CD45 ^+^ CD11b + cells, (C) CD45 ^+^ CD11b + Ly6C + Ly6G-monocytes and (D) CD45 + CD11b + Ly6C + Ly6G + neutrophils in aortas of Ang II-infused mice. (E) Representative dot plots shown for gated aortic cells of each positive population from BMT and non-BMT mice. Labels on both axes are on a log scale. *p < 0.05 (n = 5–6 per group).

Since we observed a reduction in macrophages in the aortic root of BMT mice, and macrophages have an active role in AAA formation [13], we questioned whether leukocyte recruitment was altered following BMT and Ang II infusion. To determine the effect of BMT on the early influx of inflammatory cells into the aortic wall we next quantified the myeloid cell population in the thoracic aortas of BMT and non- BMT *ApoE*^*−/−*^ mice following Ang II infusion for 5 days. The leukocyte content was quantified following enzymatic digestion and flow cytometry in surviving mice (Fig. 3E). There were two deaths from AAA rupture before day 5 and 2 aneurysms at harvest in the non-BMT 0.8 mg/kg/day Ang II group. We also found 1 aneurysm in surviving 3 mg/kg/day Ang II BMT mice. *ApoE*^*−/−*^ mice with large blood-filled aneurysms at the time of harvest were excluded from the analysis because of the confounding effect of blood cells trapped in aneurysms to the flow cytometric analysis of aortic cells.

The total number of leukocytes (CD45^+^) and CD11b + myeloid cells (Fig. 3A and B) in the aortas were significantly reduced in the 0.8 mg/kg/day Ang II BMT group compared to the 0.8 mg/kg/day Ang II non- BMT group. Because, circulating inflammatory Ly6C^hi^ monocytes rapidly infiltrate injured sites [16] and the two monocyte subsets enter the abdominal aorta at an early time point [17], we assessed the total monocyte population and found it to be substantially lower in the 0.8 mg/kg/day Ang II BMT than the corresponding non-BMT group (Fig. 3C). The number of neutrophils between BMT and non-BMT mice receiving 0.8 mg/kg/day Ang II was not significantly different (Fig. 3D). The total number of CD45 ^+^ cells, CD11b + cells and total monocytes and neutrophils were of comparable levels in the 0.8 mg/kg/day Ang II non-BMT group to the 3 mg/kg/day Ang II BMT group. The number of CD45 ^+^ cells, CD11b + myeloid cells and total monocytes and neutrophils were also significantly lower in the 0.8 mg/kg/day BMT group in comparison to the BMT mice receiving 3 mg/kg/day Ang II. Taken together, these results suggested an alteration in monocyte recruitment to the aorta following Ang II and BMT in *ApoE*^*−/−*^ mice.

**Fig. 4:**
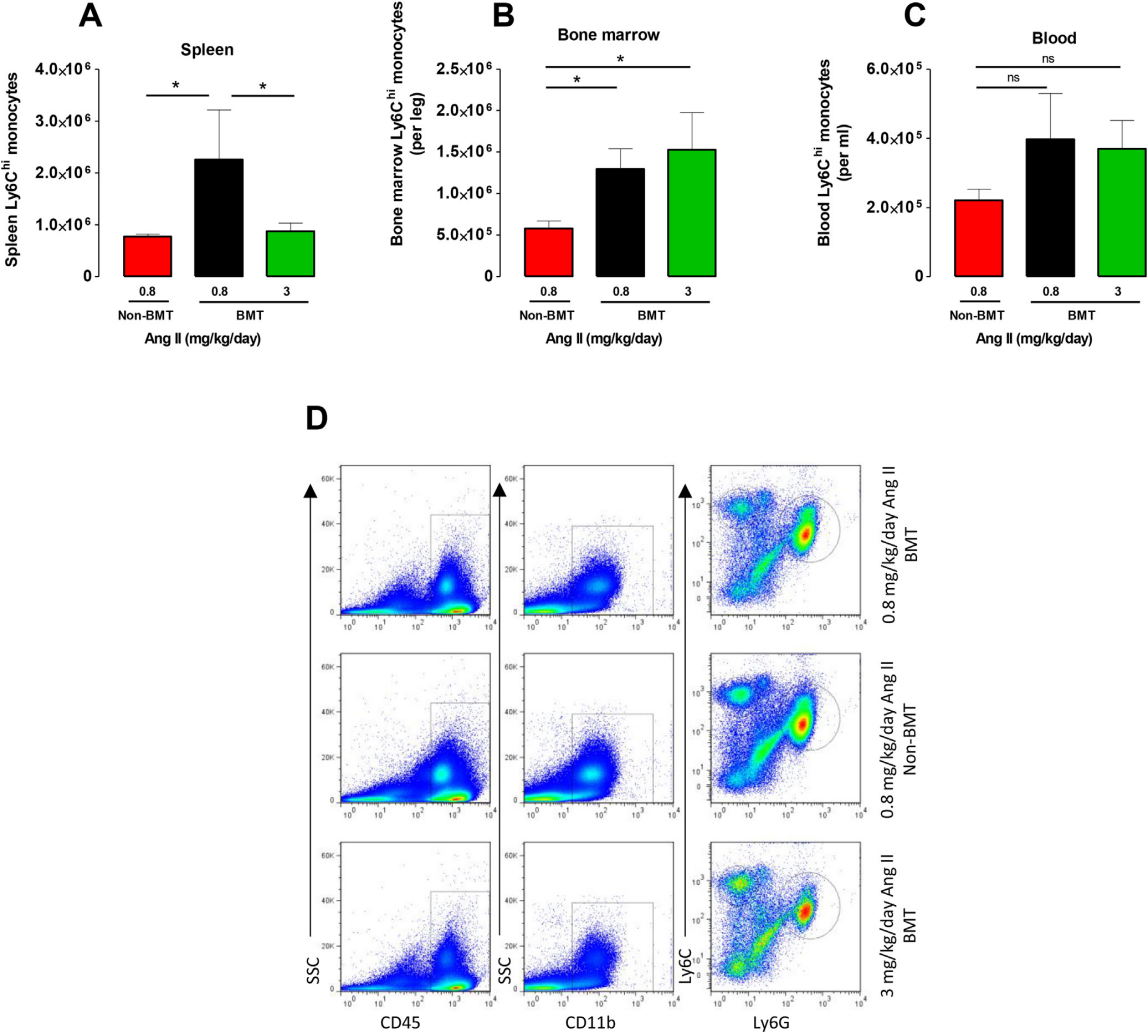
BMT affects monocyte levels in the spleen in 0.8 mg/kg/day Ang II-treated *ApoE^−^^/^^−^* mice. Flow cytometric analysis of monocytes in BMT and non-BMT mice that received Ang II infusion at 0.8 or 3 mg/kg/day for 5 days. Quantification of the numbers of CD45 ^+^ CD11b + Ly6ChiLy6G-monocytes in the (A) spleen (B) bone marrow and (C) blood of Ang II infused mice. (D) Representative dot plots shown for gated splenic cells of each positive population from BMT and non-BMT mice. Ly6Chi monocytes were identified as CD45 ^+^ CD11b + Ly6ChiLy6G-. Labels on both axes are on a log scale. *p < 0.05 (n = 5–6 per group).

Monocytes are mobilized from the bone marrow and spleen into the circulation. It has previously been shown that splenic monocytes mobilize in an Ang II–dependent manner [17,18], hence we sought to assess the numbers of monocytes in the spleen by flow cytometry (Fig. 4D). We found the number of total monocytes was significantly lower in the 0.8 mg/kg/day Ang II non-BMT than the corresponding BMT group (Fig. 4A) suggesting less monocyte mobilization in response to this dose of Ang II following BMT. This mobilization was also observed in the BMT group receiving 3 mg/kg/day Ang II. We also found an increase in the monocytes in the bone marrow of both groups that were irradiated in comparison to non-BMT mice receiving 0.8 mg/kg/day Ang II (Fig. 4B), but no differences in the number of monocytes in the blood between groups (Fig. 4C), highlighting that Ang II and BMT increases this pool of cells in the bone marrow. Taken together, these results indicate that Ang II infusion and BMT results in an reduction in aortic monocytes and possibly splenic monocyte mobilization and a substantial increase in the dose of Ang II is required to promote inflammatory cell infiltration and aneurysm formation.

## 4 Discussion

Bone marrow transplantation is an established and efficient technique to study the function of hematopoietic cells and the role of certain genes in mouse models of disease. In cardiovascular diseases, BMT experiments have provided a useful approach to elucidate the role of bone marrow derived cells and host arterial wall cells in the pathogenesis of atherosclerosis and AAA. However, the impact of BMT on leukocyte recruitment in this model has not been fully elucidated. Hence, there is a pressing need to address how BMT affects the recruitment of leukocytes and remodeling of the vascular wall in response to Ang II. We believe this is the first report of the role of BMT in the recruitment of cells to the aorta during acute vascular inflammation and aneurysm formation. We provide evidence indicating that hyperlipidemic mice that have undergone BMT have significantly reduced inflammatory responses to Ang II in the aortic root and the aortic wall, that protect these mice from the development of aortic aneurysms than non-BMT mice. For BMT studies involving the Ang II infused mouse model of AAA in *ApoE*^*−/−*^ mice, an increase in the dose of Ang II may be required to achieve the same degree of aneurysms as that of non-BMT mice.

Whilst performing BMT into irradiated *ApoE*^*−/−*^ mice, to generate bone marrow chimeric animals to assess leukocyte gene function in AAA, we observed that BMT mice infused with Ang II at 0.8 mg/kg/day did not develop aortic aneurysms in comparison with non-irradiated mice receiving this dose. The difference in AAA formation was not due to a differential hemodynamic response as the increase in blood pressure in response to Ang II was similar between groups. This is in agreement with previous studies which have demonstrated the formation of AAA in response to Ang II to be independent of changes in blood pressure [19]. The increase in blood pressure also confirmed that the Ang II was active and the lack of aneurysms in the BMT group was not due to a failure of experimental AAA model.

To further explore the finding of BMT on aneurysm formation, we carried out an Ang II dosing study in BMT mice to identify the dose that would result in a similar level of AAA to that of non-BMT mice. We found that a significant increase in the dose of Ang II to 3 mg/kg/day in BMT *ApoE*^*−/−*^ mice was required to increase aneurysm formation and atherosclerosis to the level observed in non-BMT *ApoE*^*−/−*^ mice treated with 0.8 mg/kg/day Ang II. Ang II-mediated AAA formation is driven by inflammatory cell recruitment in to the vessel wall starting from as early as day 3 of Ang II infusion, predominantly derived from the spleen [17]. At day 5 post Ang II infusion, we observed significantly reduced levels of Ly6Chi monocytes in the spleen in the non-BMT 0.8 mg/kg/day Ang II group and the BMT 3 mg/kg/day Ang II group, in comparison to the BMT 0.8 mg/kg/day Ang II group. This coincided with their simultaneous increase of total monocytes in the aorta, which may suggest that the leukocytes were mobilized from the splenic reservoir. Similarly, Mellak et al. [17] show a decrease in Ly6C^hi^ monocytes in the spleen at days 3 and 7 post Ang II infusion compared to saline in non-BMT *ApoE*^*−/−*^ mice, with their mobilization into the blood and recruitment to the aorta. Interestingly, the reduction in aortic leukocytes in the 0.8 mg/kg/day BMT group was accompanied by the increased Ly6C^hi^ monocytes in the spleen and the bone marrow. This could be accounted for by either the lack of mobilization by Ang II from these reservoirs or the effects of BMT on the numbers of cells in the bone marrow, since we observed increased levels of Ly6C^hi^ monocytes in the bone marrow of 3 mg/kg/day Ang II BMT group. Previous studies have shown that Ang II affects the compositions of hematopoietic stem cells (HSC) and myeloid progenitors by inducing hematopoietic stem and progenitor cells (HSPC) proliferation and CCR2+ monocyte accumulation in the bone marrow and spleen [20]. Although we did not analyse the HSC population in our study, it could be that the differences we have observed are due to the proliferation and differentiation of HSCs which are critical in the development of hypertension-linked vascular pathophysiology [20]. Ang II signals through Ang II type 1/2 receptors and AT1aR mRNA levels are abundantly expressed in bone marrow in comparison to AT1bR and AT2R which are not detected [21]. Furthermore, the authors show AT1aR mRNA to be down regulated following BMT, which may lead to attenuation in Ang II signalling via AT1aR in bone marrow cells. Although this was not measured in the spleen following BMT, it may suggest that BMT alters Ang II receptor levels and therefore a reduction in Ang II mediated effects such as monocyte mobilization.

It is well known that macrophages are the predominant leukocyte in Ang II induced aneurysms and originate from circulating monocytes [17,22]. This would implicate that the contribution of leukocyte infiltration to early aneurysm formation and rupture seen in our study was largely driven by the repopulated cells from the donor animal in line with studies performed by Mellak et al. [17]. However, we cannot rule out that some macrophages in the aortic wall may originate from aortic tissue-resident macrophages and this does not exclude the possibility for local proliferation of recruited monocytes or resident progenitors as other studies have shown [23]. Macrophages have both inflammatory (M1) and reparative (M2) roles in AAA through their involvement in extracellular matrix remodeling, in promotion and resolution of inflammation, and in various aspects of the tissue-healing response. As highlighted in Protti et al. BMT alters tissue macrophage polarization phenotype, with an increase in reparative M2 macrophages in the myocardium of infarcted mice [24]. This is associated with a reduced infarct size and contractile dysfunction and less remodeling following acute permanent coronary artery ligation [24]. It is well known that Ang II infusion over 14–28 days in C57 mice causes M2 macrophage accumulation in the aorta that are important in the remodeling of the vascular wall [25] and that only small numbers of cells exhibiting macrophage markers are recruited to the aorta within 3 days, significantly increasing at 7 days [26]. It would be of interest in future studies to assess the accumulation of M2 macrophages at later time points following Ang II and BMT in *ApoE*^*−/−*^ mice, given that in our current study we would not have detected M2 macrophages after 5 days of Ang II infusion.

Irradiation and BMT modifies cellular processes fundamental to aneurysm progression. We chose to focus on the early inflammatory component, however, the weakening of the arterial wall is also a major pathologic component and driven by smooth muscle cells. Our experiments are supported by other studies in which low-level laser irradiation alone attenuates aneurysm progression in the Ang II-infused *ApoE*^*−/−*^ mouse *in vivo* [27], which is linked to a modification in anti-inflammatory processes in macrophages such as inhibition of gene expression of pro-inflammatory cytokines IL-1α, IL-1β, and IL-6 and the chemoattractant CCL2 [28]. The same authors also provide contradictory effects of low-level laser irradiation on aortic smooth muscle cell proliferation, and increases in extracellular matrix protein expression and secretion [29], which are pro-aneurysmal. Taken together, this indicates the effects of irradiation are more complex, and combined with BMT in our studies may suggest both pro-proliferative and anti-inflammatory processes during the initiation and progression of Ang II driven aortic disease.

Similar studies to assess the effect of lethal total body irradiation and BMT on atherosclerosis have given contradictory results dependent on location of lesions, which may be a result of altered hemodynamics. When compared with non-BMT *LDLR*^*−*^^*/*^^*−*^ mice, BMT exacerbated high fat diet induced atherosclerosis in the aortic root, but resulted in smaller lesions in the descending aorta [4]. Studies investigating the effect of local irradiation but not BMT on carotid atherosclerosis, resulted in accelerated development of macrophage-rich, inflammatory lesions in *ApoE*^*−/−*^ mice [5,6]. In contrast, we observed a decrease in atherosclerosis progression in the aortic root after BMT in response to 0.8 mg/kg/day Ang II, with a significant reduction in macrophage content within the plaque in the BMT group. This result mirrors the findings observed in the aorta, where a decreased leukocyte influx was observed in BMT mice, indicating that the decrease in progression in atherosclerosis observed in our study was due to reduced leukocyte recruitment. It is well known that Ang II has pro-atherogenic effects and Ang II–induced lesions in *ApoE*^*−/−*^ mice are more inflammatory, predominantly made up of foam cells and lymphocytes [1].

In our current study due to the use of young, chow fed male mice, which are more resistant to the development of atherosclerosis than female mice [30], our plaques were relatively small and rich in foam cells. At this early stage of atherosclerotic plaque development, monocyte infiltration into the vessel wall is a determining factor, hence the size of our plaques may be more sensitive to changes in monocyte recruitment than larger, more complex plaques developed in previous irradiation studies from long term high fat diet feeding. Furthermore, these results are likely to be an underrepresentation of the magnitude of the response as those mice that died from AAA rupture were likely to have high levels of leukocytes in the aorta prior to aneurysm, but were not included in aortic flow cytometric and atherosclerosis analyses.

In conclusion, administration of 0.8 mg/kg/day Ang II into *ApoE*^*−/−*^ mice following BMT caused a reduction in aneurysm formation and rupture compared to non-BMT mice administered with the same dose, although systolic blood pressure remain unchanged. The protective phenotype from Ang II induced aneurysm rupture in mice that had undergone BMT is associated with a significant reduction in inflammatory monocytes in the early influx of leukocytes in the aorta. Our study demonstrates that techniques commonly used to assess murine atherosclerosis and aneurysm formation may not give the same results, and reveals the importance of examining multiple parameters of lesion and aneurysm formation. The molecular mechanisms by which irradiation and bone marrow cells have a role in Ang II infusion and vessel wall remodeling should be analysed in future studies.

## Conflicts of interest

The authors declared they do not have anything to disclose regarding conflict of interest with respect to this manuscript.

## Financial support

This work was supported by the British Heart Foundation (RG/12/5/29576), the Wellcome Trust (091504/Z/10/Z and 090532/Z/09/Z), the BHF Centre for Research Excellence (RE/13/1/30181) and the National Institute for Health Research (NIHR) Oxford Biomedical Research Centre.

## Author contributions

The study was conceived and designed by JP, GD and KMC; the experiments were performed by JP, GD, AGK and ABH.

## Appendix A Supplementary data

Supp Fig Supp Fig

## References

[1] Daugherty A., Manning M.W., Cassis L.A. (2000). Angiotensin II promotes atherosclerotic lesions and aneurysms in apolipoprotein E-deficient mice. J. Clin. Invest..

[2] de Winther M.P., Heeringa P. (2011). Bone marrow transplantations to study gene function in hematopoietic cells. Meth. Mol. Biol..

[3] Aparicio-Vergara M. (2010). Bone marrow transplantation in mice as a tool for studying the role of hematopoietic cells in metabolic and cardiovascular diseases. Atherosclerosis.

[4] Schiller N.K. (2001). Effect of gamma-irradiation and bone marrow transplantation on atherosclerosis in LDL receptor-deficient mice. Arterioscler. Thromb. Vasc. Biol..

[5] Stewart F.A. (2006). Ionizing radiation accelerates the development of atherosclerotic lesions in ApoE-/- mice and predisposes to an inflammatory plaque phenotype prone to hemorrhage. Am. J. Pathol..

[6] Hoving S. (2008). Single-dose and fractionated irradiation promote initiation and progression of atherosclerosis and induce an inflammatory plaque phenotype in ApoE(-/-) mice. Int. J. Radiat. Oncol. Biol. Phys..

[7] Robinet P. (2018). Consideration of sex differences in design and reporting of experimental arterial pathology studies-statement from ATVB council. Arterioscler. Thromb. Vasc. Biol..

[8] Henriques T.A. (2004). Orchidectomy, but not ovariectomy, regulates angiotensin II-induced vascular diseases in apolipoprotein E-deficient mice. Endocrinology.

[9] Alsiraj Y. (2017). Female mice with an XY sex chromosome complement develop severe angiotensin II-induced abdominal aortic aneurysms. Circulation.

[10] Kurtz T.W. (2005). Recommendations for blood pressure measurement in animals: summary of an AHA scientific statement from the council on high blood pressure Research, professional and public education subcommittee. Arterioscler. Thromb. Vasc. Biol..

[11] Daugherty A. (2017). Recommendation on design, execution, and reporting of animal atherosclerosis studies: a scientific statement from the american Heart association. Arterioscler. Thromb. Vasc. Biol..

[12] Galkina E. (2006). Lymphocyte recruitment into the aortic wall before and during development of atherosclerosis is partially L-selectin dependent. J. Exp. Med..

[13] Tieu B.C. (2009). An adventitial IL-6/MCP1 amplification loop accelerates macrophage-mediated vascular inflammation leading to aortic dissection in mice. J. Clin. Invest..

[14] Daugherty A. (2010). Angiotensin II infusion promotes ascending aortic aneurysms: attenuation by CCR2 deficiency in apoE-/- mice. Clin. Sci. (Lond.).

[15] Mazzolai L. (2004). Endogenous angiotensin II induces atherosclerotic plaque vulnerability and elicits a Th1 response in ApoE-/- mice. Hypertension.

[16] Raffort J. (2017). Monocytes and macrophages in abdominal aortic aneurysm. Nat. Rev. Cardiol..

[17] Mellak S. (2015). Angiotensin II mobilizes spleen monocytes to promote the development of abdominal aortic aneurysm in apoe(-/-) mice. Arterioscler. Thromb. Vasc. Biol..

[18] Swirski F.K. (2009). Identification of splenic reservoir monocytes and their deployment to inflammatory sites. Science.

[19] Cassis L.A. (2009). ANG II infusion promotes abdominal aortic aneurysms independent of increased blood pressure in hypercholesterolemic mice. Am. J. Physiol. Heart Circ. Physiol..

[20] Kim S. (2016). Angiotensin II regulation of proliferation, differentiation, and engraftment of hematopoietic stem cells. Hypertension.

[21] Fukuda D. (2008). Critical role of bone marrow angiotensin II type 1 receptor in the pathogenesis of atherosclerosis in apolipoprotein E deficient mice. Arterioscler. Thromb. Vasc. Biol..

[22] Rateri D.L. (2011). Endothelial cell-specific deficiency of Ang II type 1a receptors attenuates Ang II-induced ascending aortic aneurysms in LDL receptor-/- mice. Circ. Res..

[23] Sata M. (2002). Hematopoietic stem cells differentiate into vascular cells that participate in the pathogenesis of atherosclerosis. Nat. Med..

[24] Protti A. (2016). Bone marrow transplantation modulates tissue macrophage phenotype and enhances cardiac recovery after subsequent acute myocardial infarction. J. Mol. Cell. Cardiol..

[25] Moore J.P. (2015). M2 macrophage accumulation in the aortic wall during angiotensin II infusion in mice is associated with fibrosis, elastin loss, and elevated blood pressure. Am. J. Physiol. Heart Circ. Physiol..

[26] McNeill E. (2015). Hydrodynamic gene delivery of CC chemokine binding Fc Fusion proteins to target acute vascular inflammation in vivo. Sci. Rep..

[27] Gavish L. (2009). Low-level laser irradiation inhibits abdominal aortic aneurysm progression in apolipoprotein E-deficient mice. Cardiovasc. Res..

[28] Gavish L. (2008). Irradiation with 780 nm diode laser attenuates inflammatory cytokines but upregulates nitric oxide in lipopolysaccharide-stimulated macrophages: implications for the prevention of aneurysm progression. Laser Surg. Med..

[29] Gavish L., Perez L., Gertz S.D. (2006). Low-level laser irradiation modulates matrix metalloproteinase activity and gene expression in porcine aortic smooth muscle cells. Laser Surg. Med..

[30] Tangirala R.K., Rubin E.M., Palinski W. (1995). Quantitation of atherosclerosis in murine models: correlation between lesions in the aortic origin and in the entire aorta, and differences in the extent of lesions between sexes in LDL receptor-deficient and apolipoprotein E-deficient mice. J. Lipid Res..

