# Supplementary material for: Effect of irradiation and bone marrow transplantation on angiotensin II-induced aortic inflammation in ApoE knockout mice
Source: Atherosclerosis. 2018 Sep;276:74–82. doi: 10.1016/j.atherosclerosis.2018.07.019 (PMC6143484; doi:10.1016/j.atherosclerosis.2018.07.019)
Supplement: Supp Fig [file mmc1.pdf]

Supplementary Figure 1

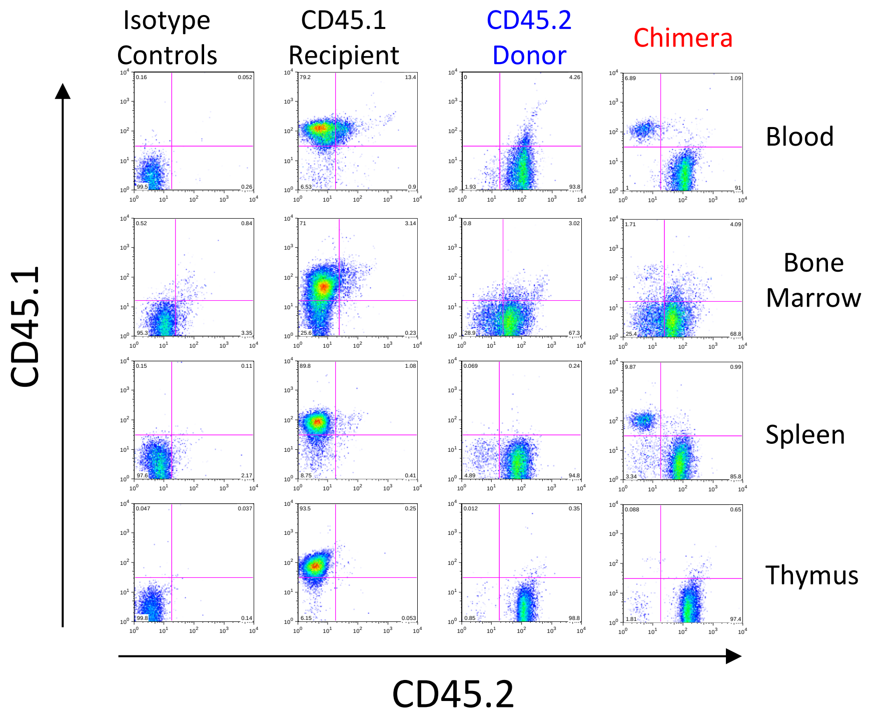

Supplementary Figure 1: Generation and Characterisation of bone marrow chimeras. CD45.1 C57bl6/J (SJL) mice were irradiated with 2 doses of 5Gy and reconstituted with bone marrow cells. Chimeric animals were culled after 6 weeks and tissues collected. (a) Single cell suspensions were produced from bone marrow, spleen and thymus and were antibody stained along side blood samples for presence of the donor CD45.2 or recipient CD45.1 allele. 25000 cells were analysed by flow cytometry to quantify reconstitution. n=2-3 (chimera) or n=6 (donor).
